# Could lessons from medical research ethics inform better conversations and governance for climate engineering research

**DOI:** 10.1038/s44168-025-00333-3

**Published:** 2026-01-19

**Authors:** Shaun D. Fitzgerald, Albert Van Wijngaarden, Ramit Debnath, Zoe Fritz

**Affiliations:** 1 Centre for Climate Repair, Department of Engineering, University of Cambridge, Cambridge, UK; 2 Scott Polar Research Institute, Department of Geography, University of Cambridge, Cambridge, UK; 3 Department of Architecture, University of Cambridge, Cambridge, UK; 4 The Healthcare Improved Studies (THIS) Institute, University of Cambridge, Cambridge, UK

**Keywords:** Environmental studies, Geography, Geography

## Abstract

Conversations about climate engineering are difficult to have in many spaces. While public debate deserves exploration, we focus on the difficulties *scientific* discussions around climate engineering face. For inspiration on how to improve this contested space we turn specifically to the history of controversial medical research. Some ways to move forward might consist of establishing an oversight mechanism, defining boundaries and introducing a specialised review system.

## Introduction

Large scale engineering interventions in the climate system—ranging from the injection of reflective particles into the stratosphere, to the artificial creation of Arctic sea ice—are controversial. And for good reasons: one concern is that climate engineering research could divert energy and resources away from tackling the underlying problem of excess levels of greenhouse gasses, and even be used as a diversion to prolong the status quo and fossil fuel economy^1^; another that even if billed as purely exploratory, research pathways might place us on a ‘slippery slope’ that will make eventual deployment far more likely^2^; a third, that climate engineering could be ungovernable, or that states or militaries could securitize such technologies; and then there are also concerns research will reflect the interests of already invested rich industrialised countries^3^.

Many of these concerns place climate engineering research into question. Beyond the above-mentioned concerns, research itself also raises a series of worries about straightforward environmental risk (in the context of outdoor experiments). However, given the current state of climate change^4^, projections about worsening emissions^5^, and an increasing interest in climate engineering – UK government funding agencies having announced a total of £67 m of funding^6,7^ and one for-profit company raising $60 m in 2025 alone^8^—there is an urgency in determining what research is appropriate, and with what regulation it might be permissible.

Conversations about climate engineering are difficult to have in many spaces, as perhaps is best illustrated by the increasing prominence of conspiracy theories in public conversation^9^. While the public debate deserves exploration, we want to focus on the difficulties *scientific* discussions around climate engineering face. We think that these have at times been complicated by a relatively polarised state, which tends to prevent constructively critical conversations from taking place. This polarisation is mostly visible in the organisation of research networks and “behind the scenes” conversations and comments, but also to a certain extent in publications. The lead article in Frontiers’ 2025 collection of articles on polar climate engineering by Siegert et al. ^10^ for example sharply contrasts with the accompanying publication by Moore et al. ^11^, similarly the call for a non-use agreement for solar climate engineering^1^ motivated a starkly contrasting response by other researchers^12^.

Climate engineering is not the only research area in which the ethics of research itself has been put into question. There is significant literature on the way similar controversial emerging technologies and research fields, ranging from infectious diseases and cloning to discussions around the development of Large Language Models and AI, or nanotechnologies, have developed, and could be governed^13^. All these fields may be able to offer insights into how to deal with the difficulties of research into climate engineering. For inspiration on how to improve the contested space of climate engineering research we turn specifically to the history of controversial medical research.

## Trying to make the debate transparent and constructive

Discussions around in vitro fertilisation (IVF) in the 1980s were polarised, preventing spaces for discussion to emerge outside siloed conversations. Following the first IVF birth there was concern and criticism from politicians, religious groups, research funders and the public in terms of the creation or use of embryos which would not go on to be fertilised. The UK Government responded to this by commissioning the Report of the Committee of Inquiry into Human Fertilisation and Embryology, chaired by Oxford philosopher Mary Warnock and assisted by development biologist Anne McLaren. In her inquiry, Warnock listened to and recognised the concerns of all of those involved. She established common language and common ground and used this to have a transparent and constructive debate^14^. Ultimately, the report made 64 recommendations including that embryos should not be kept alive for more than 14 days and concluded that *“the embryo of the human species ought to have a special status”*. Many of these recommendations are now enshrined in legislation, allowing research to continue in the context of shared ethical standards and constraints.

A more recent example—and a different approach—can be found in transplant surgery. There are an increasing number of people on dialysis and awaiting kidney transplants, while many people die from out-of-hospital cardiac arrests. Ethical questions and emotive debates exist about retrieving organs in this situation, including defining when someone’s life ends and whether it is appropriate to perfuse abdominal organs in order to preserve them, while waiting for full consent for donation^15^. A recent empirical study of 20 donors confirmed that there was no circulation to the brain in this circumstance^16^; other qualitative studies are being undertaken to understand the experiences and concerns of families of donors and of practitioners. Importantly, by carefully delineating the areas of contention and considering how they can be empirically explored, the debate has shifted towards what is known and what can be tested.

Both examples illustrate the importance of finding a common ground from which it might be possible to engage in a mutually acceptable conversation and start exploring knowledge and research in a transparent way. In the context of the current climate crisis and continued lack of climate action, ideas of climate engineering force a reckoning with extremely complicated and fundamental questions around human-environment relationships, climate justice, and the role of technology in imagined futures. Perhaps openly discussing these underlying questions can offer a point of departure for discussions on the ethics of climate engineering research. Learning from the IVF-example, the creation of a commission or institution by the government might bring some pragmatic relief to the climate engineering discussion. Learning from the transplant example, limited small scale studies in areas of contention within the field of climate engineering might be used to inform and further the scientific debate.

## Informing governance and regulation for climate engineering research

Beyond the polarised state of the debate, another significant obstacle for legitimate and acceptable climate engineering research remains the lack of dedicated governance structures. That is not for a lack of trying: many national and international reports, commissions and publications by independent groups and organisations have sought to forward governance initiatives. Notable examples of this are the development of the Oxford principles^17^, the US’s National Academies of Sciences, Engineering, and Medicine’s solar climate engineering research governance recommendations^18^, the EU’s Geo-engineering: A roadmap towards international guidelines report^19^, the American Geophysical Union’s Ethical Framework Principles^20^, and the ongoing EU-funded Co-CREATE project on solar climate engineering governance^21^.

Although some argue that current legal and governance regimes already significantly apply to climate engineering activities^22^, most researchers in the field think that specific governance for climate engineering is required, albeit that there is great disagreement around what form this should take. Again, some lessons can be learned from medical research ethics, which might serve as inspiration for climate engineering research governance.

The first international code of ethics for research on human subjects was the Nuremberg code, introduced in 1947 after the Second World War; this 10-point set of rules for the conduct of human experiments has formed the backbone of many international governance codes since. Today in the UK the Health Research Authority (HRA) oversees Research Ethics Committees which review research proposals to safeguard participants. The HRA’s National Research and Ethics Advisors’ Panel (NREAP) provides advice on policy development and emerging issues, while the independent Nuffield Council on Bioethics identifies, analyses and advises on ethical issues in biomedicine and health. Examples of guidance published by institutions such as these include analysing ‘slippery slope’ arguments relating to stem cell and embryo research, new vaccine development^23^ and gene therapy^24^, which, since 2012, has had its own Gene Therapy advisory committee.

While these organisations provide national guidance, pathogens and treatments cross borders—much like the potential impact of climate engineering—and so international controls and guidance are governed by the World Health Organisation (WHO). WHO provides strict controls on research done on smallpox^25^ and ‘gain-of-function’ research on pathogens (research to make them more virulent). The decisions on what to ban and what to limit are arrived at following expert review of evidence, consideration of consequences (intended and unintended) and planned timescales to review.

The existence and functioning of these organisations suggest that consensus on ethically challenging research is possible, if not always easy. Similar to climate engineering research, medical research governance has to deal with the multiple scales at which governance might be needed, and has to find ways to negotiate and delegate governance between national and international levels.

## A way forward?

Although it might be possible to learn lessons from the history of medical research debates and governance, we do not want to say that this is in any way easy or straightforward. On a more structural level, frameworks like Responsible Research and Innovation provide general insights into how emerging technologies might be governed justly and adequately, but there is no simple formula to transfer models from the history of one controversial research field to another. Yet, in the polarised debate around climate engineering, where governance is desired but not yet formalised, we think the concrete examples from medicine we outlined above can provide some insights and inspiration.

Synthesising the aspects identified in the previous sections, some ways to move forward might consist of:

1. **Establishing an oversight mechanism:** Establish national and international multi-disciplinary expert groups (including philosophers or ethicists, and public representatives) to oversee and regulate ethical research; this should include voices from different communities to ensure that multiple perspectives are sought in devising and updating frameworks as necessary.
2. **Defining boundaries:** Be transparent about concerns within the research community, in particular identifying ‘red lines’ which would be unacceptable to cross.
3. **Introducing a specialised review system:** Develop specialised national and international ethics committees to review research proposals, with explicit requirements for different kinds of research (e.g. within the laboratory versus outdoor experimentation, scale of experimentation, etc.).

Beyond these three points, there are many other requirements for just, fair, and acceptable research practices and governance. These include their engagement with—and responsibility towards—the public and relevant communities, and the communication and transparency of research objectives, design, and findings.

We recognise that calling for governance is not new: our goal is to focus the activity and debate by highlighting parallels with medical research. Fundamental ethical questions surrounding climate engineering should not be sidelined. We need to address them directly by creating more open spaces for thoughtful dialogue and governance. This will help us to work together to address one of the most pressing long-term issues facing humanity and the planet—the climate crisis.

## Data Availability

No datasets were generated or analysed during the current study.
